# Supplementary material for: Mortality and admissions for cerebrovascular and cardiovascular diseases after the Accumoli-Amatrice 2016 earthquake
Source: PLoS One. 2025 Jun 18;20(6):e0326242. doi: 10.1371/journal.pone.0326242 (PMC12176150; doi:10.1371/journal.pone.0326242)
Supplement: S1 File — (DOCX) [file pone.0326242.s001.docx]

**Mortality and admissions for cerebrovascular and cardiovascular diseases after the Accumoli-Amatrice 2016 earthquake**

**Supplementary materials**

**Demographic, socioeconomic characteristics and access to hospital care facilities in the group A and group B municipalities**

The comparison of demographic, socio-economic and service accessibility characteristics in the two groups was conducted by comparing their distribution by age group starting at the age of 41, considering that cardiac events are rare at lower ages. For the comparison of socio-economic characteristics, the deprivation index was used, which comprises five factors (low education, unemployment, housing overcrowding, rented households and single-parent households with minor children). This composite index is calculated with a z-score and is the index commonly used in Italy in epidemiological studies; higher values of the index correspond to higher level of deprivation (1). Finally, accessibility to hospital facilities was assessed through the road distance (in kilometres) to the nearest hospital service.

**Table S1. Age distribution by 10-year age group in residents ageing 41year old and more**

|  | | | |  |
| --- | --- | --- | --- | --- |
| **Age class** | **Group A** | **%** | **Group B** | **%** |
| 41-50 | 8,311 | 21.7 | 3,411 | 21.2 |
| 51-60 | 9,136 | 23.9 | 3,927 | 24.4 |
| 61-70 | 8,174 | 21.4 | 3,360 | 20.9 |
| 71-80 | 6,585 | 17.2 | 2,693 | 16.8 |
| 81-90 | 4,904 | 12.8 | 2,129 | 13.2 |
| 91-100 | 1,124 | 2.9 | 551 | 3.4 |
| Total | 38,234 |  | 16,071 |  |

Two-sample Wilcoxon rank-sum (Mann–Whitney) test = -1.768 (p=0.07)

**Comparison of the score of the deprivation index (in terms of z-score)**

**Figure S1.** Deprivation index values of the municipalities by group (A= most hit area; B= control group)

Group A: Mean= -1.54 (Standard Error=0.27)

Group B: Mean=-1.75 (Standard Error =0.33)

T-test = 0.41, p=0.68

**Comparison of the mean distance to the nearest hospital service**

**Figure S2.** Distance from the nearest hospital by group (A= most hit area; B= control group)

Group A: Mean= 22.4 Km (Standard Error=3.05)

Group B: Mean= 24.2 Km (Standard Error =1.41)

T-test = 0.61, p=0.54

The two groups of municipalities did not significantly differ neither for demographic and sociodemographic characteristics, nor in terms of accessibility to the nearest hospital service.

**Table S2.** List of included cardiovascular and cerebrovascular diseases and the relative coding in the ICD-9-CM and ICD-10.

| **Cardiovascular and Cerebrovascular Disease** | **ICD-9-CM Code** | **ICD-10 Code** |
| --- | --- | --- |
| Angina Pectoris | 411.1, 413.0, 413.1 | I20 |
| Acute Myocardial Infarction | 410 | I21 |
| Other acute and subacute forms of ischemic heart disease | 411.0, 416.9 | I24 |
| Cardiomyopathy | 425, 421.0 | I42 |
| Atrioventricular block and left bundle branch block | 426.0, 426.1, 426.2, 426.3 | I44 |
| Other specified conduction disorders | 426.4, 426.5, 426.7, 426.8, 426.9 | I45 |
| Cardiac dysrhythmias | 426.0, 427.4, 427.6, 427.8, 427.9 | I49 |
| Paroxysmal ventricular tachycardia | 427.0, 427.1, 427.2, 427.8 | I47 |
| Atrial fibrillation and flutter | 427.3 | I48 |
| Cardiac arrest | 427.5, 428.9, 429.9 | I46 |
| Other specified cardiac dysrhythmias | 426.0, 427.4, 427.6, 427.8, 427.9 | I49 |
| Cerebral infarction | 433.0, 433.1, 433.2, 433.3, 433.4, 433.7, 433.8, 433.9, 434.0, 434.9, 437.0 | I63 |
| Aortic aneurysm and aortic aneurysm rupture | 441.0, 441.1, 441.2, 441.3, 441.4, 441.5, 441.6 | I71 |
| Arterial embolism and thrombosis | 444.0, 444.1, 444.2, 444.8, 444.9 | I74 |
| Hypotension | 458.0, 458.1, 458.9 | I95 |
| Ischemic transient cerebrovascular attacks and related syndromes | 435.0, 435.1, 435.2, 435.3, 435.8, 435.9 | G45 |

**Table S3. Mortality rates of CCVDS per 1,000**

|  |  | **Observed rates** | | **Linear trend estimates of rates** | |
| --- | --- | --- | --- | --- | --- |
| **Period** | **Year** | **Group A** | **Group B** | **Group A** | **Group B** |
| Pre-earthquake | Sep 2011-Aug 2012 | 1.70 | 1.65 | 1.89 | 1.89 |
| Pre-earthquake | Sep 2012- Aug 2013 | 1.99 | 1.56 | 1.84 | 1.79 |
| Pre-earthquake | Sep 2013- Aug 2014 | 1.88 | 1.64 | 1.62 | 1.54 |
| Pre-earthquake | Sep 2014- Aug 2015 | 1.82 | 1.96 | 2.15 | 2.03 |
| Pre-earthquake | Sep 2015 - Aug 2016 | 2.23 | 1.75 | 1.74 | 1.58 |
| Post-earthquake | Sep 2016 - Aug 2017 | 2.10 | 1.90 | 2.08 | 1.79 |

Estimated average effect (AVE) of Difference-in-Difference (DID) analysis pre vs post earthquake in the Group A. AVE = 2.14; 95% Confidence Interval: 0.47-4.75

**Table S4. Mortality rates of IMA per 1,000**

|  |  | **Observed rates** | | **Linear trend estimates of rates** | |
| --- | --- | --- | --- | --- | --- |
| **Period** | **Year** | **Group A** | **Group B** | **Group A** | **Group B** |
| Pre-earthquake | Sep 2011-Aug 2012 | 0.93 | 0.74 | 0.85 | 0.85 |
| Pre-earthquake | Sep 2012- Aug 2013 | 0.94 | 0.87 | 0.95 | 0.91 |
| Pre-earthquake | Sep 2013- Aug 2014 | 1.06 | 0.73 | 0.84 | 0.76 |
| Pre-earthquake | Sep 2014- Aug 2015 | 0.95 | 0.75 | 0.93 | 0.81 |
| Pre-earthquake | Sep 2015 - Aug 2016 | 1.12 | 0.81 | 0.94 | 0.78 |
| Post-earthquake | Sep 2016 - Aug 2017 | 1.13 | 0.80 | 1.05 | 0.80 |

Estimated average effect (AVE) of Difference-in-Difference (DID) analysis pre vs post earthquake in the Group A. AVE=1.70; 95% Confidence Interval: 1.53-1.87

**Table S5. Hospital rates of CCVDS per 1,000**

|  |  | **Observed rates** | | **Linear trend estimates of rates** | |
| --- | --- | --- | --- | --- | --- |
| **Period** | **Year-Month** | **Group A** | **Group B** | **Group A** | **Group B** |
| Pre-earthquake | 2014-10 | 29.25 | 18.03 | 26.28 | 26.28 |
| Pre-earthquake | 2014-11 | 13.66 | 20.33 | 17.15 | 17.09 |
| Pre-earthquake | 2014-12 | 15.25 | 25.88 | 21.05 | 20.93 |
| Pre-earthquake | 2015-01 | 25.39 | 24.22 | 24.48 | 24.31 |
| Pre-earthquake | 2015-02 | 20.91 | 20.32 | 21.31 | 21.08 |
| Pre-earthquake | 2015-03 | 34.42 | 17.76 | 29.73 | 29.44 |
| Pre-earthquake | 2015-04 | 19.48 | 18.27 | 18.40 | 18.05 |
| Pre-earthquake | 2015-05 | 18.45 | 20.84 | 18.95 | 18.54 |
| Pre-earthquake | 2015-06 | 16.70 | 19.78 | 16.52 | 16.05 |
| Pre-earthquake | 2015-07 | 25.24 | 22.32 | 21.76 | 21.23 |
| Pre-earthquake | 2015-08 | 18.09 | 14.29 | 15.86 | 15.28 |
| Pre-earthquake | 2015-09 | 21.20 | 26.78 | 24.23 | 23.59 |
| Pre-earthquake | 2015-10 | 22.88 | 16.94 | 18.82 | 18.11 |
| Pre-earthquake | 2015-11 | 34.44 | 25.21 | 30.63 | 29.87 |
| Pre-earthquake | 2015-12 | 20.22 | 16.92 | 18.50 | 17.68 |
| Pre-earthquake | 2016-01 | 20.00 | 18.20 | 18.51 | 17.64 |
| Pre-earthquake | 2016-02 | 14.48 | 14.53 | 15.01 | 14.08 |
| Pre-earthquake | 2016-03 | 23.74 | 21.02 | 22.46 | 21.47 |
| Pre-earthquake | 2016-04 | 12.60 | 23.48 | 17.43 | 16.38 |
| Pre-earthquake | 2016-05 | 18.00 | 16.27 | 17.88 | 16.77 |
| Pre-earthquake | 2016-06 | 38.49 | 24.08 | 31.95 | 30.78 |
| Pre-earthquake | 2016-07 | 22.28 | 13.21 | 18.51 | 17.28 |
| Pre-earthquake | 2016-08 | 23.92 | 14.35 | 19.13 | 17.84 |
| Post-earthquake | 2016-09 | 23.78 | 12.57 | 27.05 | 10.07 |
| Post-earthquake | 2016-10 | 21.12 | 15.06 | 24.51 | 9.41 |
| Post-earthquake | 2016-11 | 31.76 | 12.35 | 32.72 | 19.51 |
| Post-earthquake | 2016-12 | 23.93 | 14.03 | 23.62 | 12.28 |
| Post-earthquake | 2017-01 | 58.59 | 29.86 | 50.78 | 41.33 |
| Post-earthquake | 2017-02 | 27.81 | 14.80 | 28.87 | 21.30 |
| Post-earthquake | 2017-03 | 14.09 | 15.50 | 21.34 | 15.65 |
| Post-earthquake | 2017-04 | 17.52 | 18.98 | 20.32 | 16.51 |
| Post-earthquake | 2017-05 | 19.68 | 14.69 | 16.57 | 14.65 |
| Post-earthquake | 2017-06 | 18.85 | 18.14 | 17.88 | 17.84 |
| Post-earthquake | 2017-07 | 21.64 | 16.70 | 15.97 | 17.81 |
| Post-earthquake | 2017-08 | 11.26 | 11.03 | 6.16 | 9.88 |

Estimated average effect (AVE) of Difference-in-Difference (DID) analysis pre vs post earthquake in the Group A. AVE=6.72; 95% Confidence Interval: -19.04-32.48

**Table S6. Hospital rates of IMA per 1,000**

| **Period** | **Year-Month** | **Group A** | **Group B** | **Group A** | **Group B** |
| --- | --- | --- | --- | --- | --- |
| Pre-earthquake | 2014-09 | 1.89 | 2.09 | 3.00 | 3.00 |
| Pre-earthquake | 2014-10 | 3.17 | 5.47 | 4.15 | 3.92 |
| Pre-earthquake | 2014-11 | 3.00 | 6.65 | 5.17 | 4.72 |
| Pre-earthquake | 2014-12 | 1.98 | 4.05 | 3.24 | 2.56 |
| Pre-earthquake | 2015-01 | 5.76 | 3.10 | 4.67 | 3.77 |
| Pre-earthquake | 2015-02 | 5.94 | 3.94 | 5.69 | 4.56 |
| Pre-earthquake | 2015-03 | 1.34 | 2.29 | 3.12 | 1.77 |
| Pre-earthquake | 2015-04 | 3.34 | 3.37 | 3.87 | 2.29 |
| Pre-earthquake | 2015-05 | 5.81 | 4.53 | 5.83 | 4.03 |
| Pre-earthquake | 2015-06 | 2.21 | 2.65 | 2.58 | 0.56 |
| Pre-earthquake | 2015-07 | 4.05 | 4.72 | 4.94 | 2.69 |
| Pre-earthquake | 2015-08 | 3.22 | 2.43 | 3.96 | 1.49 |
| Pre-earthquake | 2015-09 | 5.65 | 2.17 | 4.85 | 2.15 |
| Pre-earthquake | 2015-10 | 2.76 | 3.19 | 4.10 | 1.17 |
| Pre-earthquake | 2015-11 | 4.68 | 2.81 | 5.38 | 2.23 |
| Pre-earthquake | 2015-12 | 1.76 | 5.57 | 5.84 | 2.47 |
| Pre-earthquake | 2016-01 | 2.71 | 4.94 | 5.66 | 2.06 |
| Pre-earthquake | 2016-02 | 2.41 | 2.48 | 4.36 | 0.54 |
| Pre-earthquake | 2016-03 | 8.60 | 10.97 | 12.23 | 8.18 |
| Pre-earthquake | 2016-04 | 1.53 | 6.35 | 6.03 | 1.76 |
| Pre-earthquake | 2016-05 | 2.06 | 3.01 | 5.15 | 0.65 |
| Pre-earthquake | 2016-06 | 16.18 | 2.34 | 17.13 | 12.41 |
| Pre-earthquake | 2016-07 | 3.97 | 2.61 | 5.87 | 0.92 |
| Pre-earthquake | 2016-08 | 9.92 | 3.23 | 9.22 | 4.05 |
| Post-earthquake | 2016-09 | 4.15 | 2.54 | 7.08 | 0.10 |
| Post-earthquake | 2016-10 | 2.00 | 1.99 | 5.38 | 0.00 |
| Post-earthquake | 2016-11 | 4.95 | 3.05 | 7.69 | 1.19 |
| Post-earthquake | 2016-12 | 10.93 | 4.79 | 10.93 | 4.79 |
| Post-earthquake | 2017-01 | 24.55 | 2.07 | 17.54 | 11.75 |
| Post-earthquake | 2017-02 | 4.43 | 3.23 | 6.84 | 1.41 |
| Post-earthquake | 2017-03 | 3.89 | 7.28 | 9.52 | 4.45 |
| Post-earthquake | 2017-04 | 2.57 | 3.38 | 5.68 | 0.96 |
| Post-earthquake | 2017-05 | 7.26 | 2.45 | 6.50 | 2.13 |
| Post-earthquake | 2017-06 | 7.41 | 6.18 | 8.62 | 4.61 |
| Post-earthquake | 2017-07 | 6.34 | 2.98 | 6.18 | 2.52 |
| Post-earthquake | 2017-08 | 2.83 | 2.32 | 3.47 | 0.17 |

Estimated average effect (AVE) of Difference-in-Difference (DID) analysis pre vs post earthquake in the Group A. AVE=2.91; 95% Confidence Interval: -5.94-11.77

**REFERENCES**

1. Rosano A, Pacelli B, Zengarini N, Costa G, Cislaghi C, Caranci N. Aggiornamento e revisione dell’indice di deprivazione italiano 2011 a livello di sezione di censimento. E&P. 2020 Jun;44(2–3):162–70.
