# Supplementary material for: Mortality and admissions for cerebrovascular and cardiovascular diseases after the Accumoli-Amatrice 2016 earthquake
Source: PLoS One. 2025 Jun 18;20(6):e0326242. doi: 10.1371/journal.pone.0326242 (PMC12176150; doi:10.1371/journal.pone.0326242)
Supplement: Attachment — Submitted filename: point-by-point response_rev_2.docx [file pone.0326242.s003.docx]

**Reviewer 2**

**I would like to thank you for providing me the opportunity to read and provide a review of this paper.
I've found interest in reading the paper even if the research question explored is not particularly new given the existing literature on earthquakes and CVDs worldwide (e.g. Japan and New Zealand).**

We really thank the reviewer for the valuable suggestions. We are very grateful for their appreciation and their precious comments, that we strictly followed.

**I have a major concern regarding the statistical approach adopted by the authors.
If I got it right, the methodology adopted by the authors is basically a before/after comparison of those individuals with residence in the 17 selected municipalities that were partially or totally affected by the Accumoli-Amatrice earthquake. The control period considered is basicallly the pre-earthquake period: from August 24, 2014 to August 23, 2016.
However this approach, as clearly discussed by the authors, is subject to some relevant limitations. In particular, results may be biased by “additional risk factors for AMI” such as “[…] unusual cold registered on January after the earthquake which could have contributed to a part of hospitalizations and/or deaths and the inconvenient home conditions of many people after the event, i.e. camping tends, caravans and containers, which could have caused further stress and impact on the cardiovascular wellbeing”.
However, these kind of biases may be eliminated if the authors would have adopted a different econometric technique among those belonging to the Counterfactual Impact Evaluation (CIE) family that better suits the research question investigated (for example a Difference in Difference model?). Including also data on individuals that were resident in municipalities closed to the earthquake but that were not affected by it because they were not in proximity of the epicentre as control group -for example- may help in taking into account any simultaneous risk factor that may have affected the population in the considered periods before and after the sudden earthquake event.
I wonder why the authors limited their analysis to the simple before/after approach while other CIE tecniques would better fit in this context. I haven't found any discussion about it in the actual version of the paper while I personally think that the choice of the appropriate identification strategy is crucial in this context.**

We thank the reviewer for the valuable comment. Following your kind suggestion, we decided to adopt the suggested counterfactual impact evaluation approach. For this aim we collected relevant data also from a group of municipalities in the area surrounding the epicentre of the earthquake but with limited effects compared to the most hit area, previously selected. Hence the analysis was conducted through Difference-in-Differences (DID) regression models, mitigating the effects of extraneous factors (e.g. cold temperatures and other environmental factors) and selection bias. The results of the DID regression confirmed the increase in the post-earthquake period of the occurrence of Acute myocardial infarction in the most hit area compared with the control group of municipalities.
