# Supplementary material for: Mortality and admissions for cerebrovascular and cardiovascular diseases after the Accumoli-Amatrice 2016 earthquake
Source: PLoS One. 2025 Jun 18;20(6):e0326242. doi: 10.1371/journal.pone.0326242 (PMC12176150; doi:10.1371/journal.pone.0326242)
Supplement: Attachment — Submitted filename: Response to Reviewers_round_2.docx [file pone.0326242.s004.docx]

**I agree with the previous reviewer 2’s comment, and changing the overall analyses using the DID framework seem reasonable. Major assumption of the DID analysis is parallel trend (as authors well indicated), and authors have evaluated it using statistical testing. However, basic evaluation of the assumption can be done with simple plotting of Group A and B (such as in Figure 4).**

**In the Figures 4a 4b 6b, we can clearly see the increase in hospitalization rate in Group A even before the earthquake event (2016 06, 07, 08, 09). How could authors explain these issues? Even a statistical testing for the common trend assumption shows marginally significant results, indicating that the potential difference of the trend before the earthquake event between Groups A and B. If authors shorten their analysis period for the common trend testing, I am bit worried that they might have significant p-values. I believe reasonable and clear explanations are needed for this issue before conducting DID analysis.**

We thank the review for the valuable comment. The observed trend can be attributed primarily to the natural aging of the population in Group A, which led to a gradual increase in hospitalizations over time. This demographic shift, independent of the earthquake event, is a well-documented factor influencing hospitalization rates.

Additionally, regarding the peak observed in June 2016, we note that this period was characterized by an abnormal rise in temperatures due to a heat wave, which likely contributed to an increase in hospital admissions, particularly among the elderly population (<https://www.ansa.it/english/news/general_news/2016/07/08/heatwave-in-italy-set-to-intensify_0a21cf60-582f-415a-8e7e-140b69448783.html>). Heat waves are known to disproportionately affect vulnerable individuals, leading to higher rates of hospitalization for conditions such as dehydration, cardiovascular complications, and respiratory distress (e.g., Zhou M, Zhao G, Zeng Y, Zhu J, Cheng F, Liang W. Aging and cardiovascular disease: current status and challenges. Reviews in Cardiovascular Medicine. 2022 Apr 8;23(4):135).

We added a paragraph to provide explanations about this issue. Please, see lines 318-322.

**More explanation for the biological plausibility of authors’ findings are needed. Current explanation in the introduction section focused mainly on the acute association (line 61-67, increase of stress right after the earthquake). Authors should explain why there are some lagged effects, and the effects of earthquake lasted for several months. Is it because of the long-lasting aftershocks? (BTW, I cannot see the figure 2 and hard for me to check the frequency of the aftershocks.)**

We appreciate the reviewer’s insightful suggestion to further elaborate on the biological plausibility of the observed lagged effects of the earthquake on cardiovascular outcomes. As noted, our initial explanation focused primarily on the acute adrenergic response to the main tremor and immediate aftershocks. Nonetheless, it is known that earthquake-related stressors can exert prolonged effects on cardiovascular health.

A key factor contributing to the persistence of these effects is the prolonged seismic activity (up to January 2017), as the earthquake was followed by a series of aftershocks that continued for several months, as illustrated in Figure 2. This ongoing seismic activity likely sustained heightened stress levels in the affected population, leading to repeated activation of the sympathetic nervous system and prolonged exposure to elevated catecholamines. Such chronic stress responses have been associated with sustained increases in blood pressure, endothelial dysfunction, and an increased risk of cardiovascular events over time.

We clarified these aspects in the manuscript. Please, see lines 65-69.

**To increase the possibilities of causal interpretation, I suggest authors to add some negative control analysis by using different intervention timing (some random point before the earthquake) or disease outcomes irrelevant to earthquake exposure.**

Thank you for the valuable suggestion. Unfortunately, conducting such an analysis is not feasible due to data availability constraints, primarily related to privacy regulations that limit access to additional control data.

Moreover, it would be more appropriate to use years that are closer in time to the earthquake in order to minimize the influence of other confounding factors.

Notwithstanding, we believe that the robustness of our findings is supported by the consistency of the observed effect across multiple outcomes. Specifically, the impact of the earthquake on acute myocardial infarction is evident not only in hospitalization data but also in mortality records. Furthermore, our combined analysis of hospitalizations and deaths reinforces the plausibility of a true effect rather than a spurious association.

**Precise referencing is needed. For example: line 59-60, 323-334, and many others.**

Thank you for the precious suggestion. In response, we added the Wittstein et al. (2005) reference into the introduction to support the “fight or flight” adrenergic mechanism. Regarding the discussion section, the paragraph in question presents our own interpretation and primary implications of the study’s findings rather than a synthesis of previously published data; hence, we believe no additional reference is necessary there.

**Current table 1 is not showing the results of the DID analysis. Please include tables showing the pre-earthquake rate, post-earthquake rate and DID estimates (main findings of this study). There should be tables corresponding to Figure 3-6 (please consider using the supplementary material).**

We thank the reviewer for this suggestion. We have added to the Supplementary Material detailed tables showing the distribution of the pre‐ and post‐earthquake rates of mortality and hospitalizations.

We would like to clarify that Table 1 was not intended to display the results of the DID analysis; rather, it reports the overall rates for the two outcomes (hospitalization and death for AMI) as aggregated summary statistics, and it has been updated accordingly. In addition, because the overall AMI data (i.e., deaths + hospitalizations) were available on an annual basis only and comprised a limited number of observations, a DID approach was not feasible for these combined data. Instead, we employed an alternative analytic strategy using the chi‐square test to assess differences in these rates.

**Please add more explanation for selecting different time frame for mortality and hospitalization. Authors presented it was based on the power calculation but not sure how this can be applied to the DID framework (Not familiar with DID studies using it). In addition, please change reference 15 16 to journal paper if it is possible. Line 121-124 needs more detailed explanation for starting date, end date, number of shelters, and others.**

Thank you for raising these points. To clarify, the power calculation was performed using the relative risks observed in the pre‐ and post‐earthquake periods, and these calculations remain valid for our DID framework. In other words, the choice of different time frames for mortality and hospitalization outcomes was guided by these power considerations, ensuring that our analyses had sufficient statistical power to detect a meaningful effect in each case.

Regarding references number 15 and 16, these are primary institutional Italian sources that provided the necessary data for our expected values calculations. Unfortunately, journal papers reporting the same data are not available at this time.

Furthermore, we would like to clarify that the starting and ending dates of the study period were chosen to capture the window in which adverse post‐earthquake conditions—such as makeshift shelter accommodations (e.g., tents and cars) and extreme weather events like cold waves—were most likely to contribute to stress‐induced CCVD events. Although we agree that information on the number of shelters and similar specific parameters would further inform the context, such data were not available to us. In the manuscript, the examples provided (i.e., makeshift accommodations and cold waves) are intended to illustrate potential environmental stressors rather than to serve as quantitative inputs. We have now clarified in the revised text that while we cannot specify the exact number of shelters or related details due to data limitations, these examples underscore the plausibility of a prolonged period of adverse living conditions that could have increased the risk of CCVD events.

**Detailed numbers of the DID analysis could be added in the Results section**

Thank you for the valuable suggestion. We added the estimated values for the outcome whose difference in the pre- and post-period resulted to be statistically significant.

**line 130 Figure 1. Current Figure 1 is hard to see and presents limited information. (Not sure why authors used different colored marks: Green Yellow, Red, Blue) I suggest authors to use map figure with additional information regarding earthquake epicenter.**

Thank you for your precious suggestion. We revised Figure 1 by adding an earthquake epicenter. The different colored marks are needed to identify municipalities that belong to the several counties hit by the earthquake. Figure 1’s legend reports the list of municipalities for each marker.

**line 132 Authors mentioned similar characteristics, did not indicate what kinds of characteristics are similar. Is it population? Income? Number of hospitals? These factors should be similar across Group A and B, but they are not presented in the manuscript. Maybe supplementary table can be used here.**

The groups of municipalities A and B are similar in terms of demographic structure, socio-economic conditions and accessibility of care facilities. In the supplementary material, data and information on these aspects were added by conducting appropriate comparison tests

**Can’t see the Figure 2.**

Thank you for pointing out this issue. We replaced the older file and uploaded the final version of Figure 2.

**Line 205-206 Not sure how the authors included gender and age information in the regression model instead of conducting stratification analysis. Please clarify (maybe using the equation?)**

Actually, we considered age and gender as possible confounding factors. This aspect was specified in the methods section. Anyway, as suggested, we corrected the equation of the model including the two variables (age and gender). Please, see lines 205-206.

**Line 216-218 Other region-specific time-varying (e.g. monthly temperature for hospitalization analysis) variable can also include in the DID model. Not sure why authors did additional analyses using T-test.**

We thank the reviewer for the comment. In our study, we examined whether regional differences in temperature might affect the outcomes. However, our analysis of the temperature data revealed that there was no substantial variability in the monthly temperatures between areas of Group A and B. For this reason, temperature was not included as an additional time-varying covariate in the DID model. The t-test was specifically employed to test the difference between the mean temperatures in the two areas, and the results confirmed that there was no statistically significant difference.

**Figures 3 and 4 are not self-explanatory and hard to interpret. Please add figure titles to each figure. In addition, Figure 6b shows values below 0. Why is that?**

We thank the reviewer for these helpful suggestions. Regarding Figures 3 and 4, we note that their titles were included in the text; however, based on the reviewer’s comment, we have now revised the figures to include explicit titles and clear axis labels to enhance their self-explanatory nature. As for Figure 6b, the original model produced some values below 0. We have since revised the model by imposing a constraint that the rates cannot be less than 0. This update ensures that all output values are non-negative, consistent with the underlying epidemiological meaning. Please, see the revised Figures.
