# Supplementary material for: Mortality and admissions for cerebrovascular and cardiovascular diseases after the Accumoli-Amatrice 2016 earthquake
Source: PLoS One. 2025 Jun 18;20(6):e0326242. doi: 10.1371/journal.pone.0326242 (PMC12176150; doi:10.1371/journal.pone.0326242)
Supplement: Attachment — Submitted filename: Response to Reviewers.docx [file pone.0326242.s006.docx]

**The work, after the second revision, shows significant improvements in terms of scientific robustness and clarity, but still has some aspects to be refined for optimal publication. Strengths include better justification of the use of the Difference-in-Differences (DID) model, with tests for the assumption of parallel trends and more detailed explanations of the results; the addition of additional data that improve transparency; and effective clarifications on biological plausibility and pre-earthquake trends, supported by bibliographic references. The inclusion of age and gender in the model and the analysis of regional temperatures help reduce bias, while originality lies in filling a gap in the Italian literature with public health implications.**

Thank you for your thoughtful and detailed assessment of our revised manuscript. We greatly appreciate your recognition of the improvements made regarding the scientific robustness and clarity of our work. We look forward to addressing the remaining aspects you have identified to further optimize our manuscript for publication.

**Remaining critical points include limitations due to the use of aggregate data, which reduces granularity and increases the risk of ecological bias;**

We thank the Editor for the valuable suggestion. The use of aggregated data was unavoidable because of restrictions due to privacy regulations concerning health data for academic researchers in our country. This limitation has been reported in the discussion and cannot be overcome. Please, see lines 410-419.

**the absence of a negative control analysis, which weakens causal inference despite the authors' justification;**

Thank you for the comment. This limitation has been added in the revised version. We also included data at country level showing that both mortality rates for CVD and hospitalization for AMI steadily decreased in the studied periods. Please, see lines 431-435.

**and the difference in pre-earthquake trends between groups, with marginal significance of the test for parallel trends (p=0.07 for AMI), which raises doubts about the validity of the DID.**

We thank the Editor for the precious comment. The test for parallel trends was largely not significant for CVD, both for mortality (p=0.15) and for hospitalization (p=0.75), while for AMI the p-values, even though not significant, are smaller: p=0.09 for mortality and p=0.07 for hospitalization. On the other hand, the graphical test did not indicate a clear parallel trend before the earthquake only for hospitalization rates for AMI, but the statistical tests supported the hypothesis that parallel trend assumption is not violated. As a result, we can be quite confident of the validity of the DID. For sake of clarity, we added a note on this aspect among the limits. Please, see lines 442-447

**The lack of direct measurement of confounders such as socioeconomic status remains a limitation,**

The quasi- experimental design adopted with the diff-in-diff analysis “in itself” guarantees the minimization of confounding . Notwithstanding, socioeconomic status could affect the outcome differently in the treatment and control groups. However, socioeconomic status was one of the criteria adopted to select the control group (see appendix, hence an indirect measurement of socioeconomic status was adopted. As suggested by the Editor, we included in the study’s limitations the failure to directly measure the potential confounding of socio-economic status. Please, see lines 406-409.

**as does the not always intuitive presentation of the figures**

Thank you for the comment. In the revised version we have tried to improve the presentation of the figures adding details and explanations. Please, see modified figures.

**and the absence of quantitative details on the selection criteria for control municipalities, which reduces reproducibility.**

Thank you for the observation. Quantitative details on the selection criteria were reported in the appendix and referred to in the text in the “study design” section. Briefly, the selection of control municipalities was conducted on the basis of demographic characteristics, socioeconomic level and access to hospital care services. Please, see the modified document.

**The work compares with the international literature by being consistent, but less detailed in pathophysiological mechanisms and with less granular data than Japanese studies.**

We thank the editor for this constructive feedback. We agree that our study, while consistent with international literature, provides less detailed pathophysiological mechanisms compared to some Japanese studies. We would like to note that the pathophysiological mechanisms were pointed out in the introduction section of our paper.

Japanese research on earthquake-related cardiovascular events has benefited from their extensive experience with seismic events and well-established disaster medicine infrastructure.

To address these limitations, we have expanded our discussion section to include more acknowledgment of the data granularity and pathophysiological mechanisms. We have added an explicit statement addressing these specific limitations in the manuscript, highlighting both the constraints of our data collection and the contextual differences between Italian and Japanese healthcare systems and disaster response infrastructure. Please, see lines 461-467.

**In conclusion, while solid and deserving of publication, especially for the Italian context, we recommend adding an explicit statement of limitations, improving clarity of figures, and better discussion of pre-earthquake heterogeneity, possibly considering journals more specialized in disaster medicine or cardiology.**

We appreciate the editor’s recognition of our work's value, particularly for the Italian context. We have addressed all three recommendations. First, we have expanded our limitations section to provide a more comprehensive and explicit statement of the study’s constraints. Second, we have improved the clarity of our figures by enhancing their resolution, standardizing the presentation format, and adding more descriptive captions. Third, we have added a discussion of pre-earthquake heterogeneity between the two groups, acknowledging potential confounding factors and how they might have influenced our results. Following your valuable suggestion, we have incorporated additional references from specialized journals in disaster medicine and cardiology to better contextualize our findings within these specific fields. While the pathophysiological mechanisms were already addressed in our introduction and a detailed exploration is beyond the scope of our study (which focuses on evaluating mortality and hospital admissions for CCVDs in the aftermath of the 2016 Accumoli-Amatrice earthquake), these references provide valuable comparative perspectives from international disaster responses, particularly enhancing the clinical relevance of our findings. Regarding pre-earthquake heterogeneity, we have addressed this important issue in our revised manuscript. Please, see lines 467-482.
